# The Treatment of Congenital Club Foot in Infancy

**Published:** 1896-09-19

**Authors:** John Poland

**Affiliations:** Surgeon to the Miller Hospital, Greenwich, and to the City Orthopædic Hospital


### MEDICAL PROGRESS AND HOSPITAL CLINICS.

*[The Editor will be glad to receive offers of co-operation and contributions from members of the profession. All letters should be addressed to* THE EDITOR, AT THE OFFICE, 428, STRAND, LONDON, W.C.]

### THE TREATMENT OF CONGENITAL CLUB FOOT IN INFANCY.

By JOHN POLAND, F.R.C.S., Surgeon to the Miller Hospital, Greenwich, and to the City Orthopædic Hospital.

The question is so often asked when should the treatment of congenital talipes equino varus be commenced, that I have no hesitation in giving my own experience in the matter. Even now many works on surgery teach that treatment should not be attempted until the child begins to walk, but, beside the loss of valuable time, there is this objection, that the astragalus and other bones, at this tender age mostly composed of yielding cartilage, as well as the soft ligamentous structures, are allowed to retain their abnormal conditions and to still further develop the deformity. The cartilaginous bones become more ossified and augment the misshapen forms and positions on the one hand, while on the other the ligaments and fascial structures become more contracted, shortened, resisting, and rigid, rendering the deformity much more intractable. Other surgeons recommend delay until six weeks or two months of age. I am decidedly of opinion that the treatment of this form of club foot should be begun within a few hours after birth. It is an arrest of the fœtal unwinding of the limbs and affects all the structures of the foot, to some extent those of the leg and knee, and, in severe cases, those of the thigh also. In mild degrees of the deformity the mother or nurse may, under the supervision of the surgeon, do all that is requisite to effect a cure. Two or three times a day the foot should be grasped with the sole in the palm of the hand, everted, rotated at the mid-tarsal joint, abducted and flexed into its normal attitude. A mechanical appliance or surgical boot ought then to be fitted to maintain the corrected position. But in the greater proportion of cases, after correction of the distortion, the frequent application of a flexible tin splint and flannel bandage must be employed by the surgeon for some months. Plaster of Paris, glue, and other forms of stiff bandage are extensively used by some, and prove at times of advantage in the less severe degrees; but in extreme cases, where they have been used for many months consecutively, I think they lead to atrophy of the muscles of the leg from disuse. The flexible tin splint is applied daily to the outer side of the leg and foot, and gradually bent until the inversion is entirely overcome. Massage and bathing are valuable adjuncts to treatment. It is quite exceptional to find division of the tibial tendons or plantar fascia necessary to correct the varus position at this early age. An infant's foot is exceedingly pliable, and will readily yield to the gentle methods just mentioned. The varus should, if anything, be over-corrected, the anterior part of the foot being brought into the valgus or abducted position. The remaining equinus is then easily removed by division of the tendo Achilles at the age of a few months. This is the only tendon usually requiring division, but the operation should not be attempted until the varus is completely remedied, otherwise the greatest difficulty will be experienced in treatment. The tendo Achilles acts as a counter force or *point d'appui* for stretching the plantar fascia, ligaments and other structures on the inner side of the foot. This tenotomy is most usually done when the child is able to stand, or ready to walk, that is, when the limits of advantage to be gained by manipulation and mechanical means have been reached. After the division the foot should be restored (1) immediately, or (2) after a short interval to its proper position. By the first method a considerable space is left at once between the divided ends of the tendon; by the second, a slight space only for a few days, or even a week. The tendon is always reproduced—there need be no fear of non-union. A light plaster of Paris splint is then applied, and renewed once a week, or less often, or a suitable mechanical apparatus may be employed directly after the operation. A mechanical retentive appliance to the foot and leg is requisite for after treatment during the first few years of walking life—until the astragalus presents a normal facet, and the other tarsal bones develop their normal shapes under the pressure of their new and corrected positions. After tenotomy there is not infrequently in severe cases a residual and persistent tendency for the foot and leg to become inverted or everted at the knee joint. This necessitates the extension of the apparatus upwards to the knee and thigh, with a free joint at the knee, and a hip-band to ensure the foot retaining its rectified attitude. I am strongly of opinion that even in extreme cases there ought never to be any necessity for the severe operations which have so frequently to be practised in the later years of youth to obtain complete replacement, viz., tarsectomy, excision of cuboid or astragalus, linear osteotomy of tarsus, fracture of tarsus, osteotomy of tibia and fibula. Relapsed or neglected distortions of the feet are, unfortunately, too frequently seen in orthopædic hospitals, and many are due, I am convinced, to want of exact appreciation of the characteristics of the deformity. They would never be seen if treatment were commenced immediately after birth, and great care exercised in each instance. By the time it is old enough to walk the child should have an almost normally shaped foot. In the more severe instances there may be some diminution in the size of the heel and perhaps of the calf also. The method above recommended will have completed a cure just when the old form of treatment would be commenced, and the child will recollect nothing in after years of its deformed condition. It is not so much a question of mechanical apparatus or of tenotomy, but rather as I have said of exact appreciation of the characteristics and conditions which each case presents at the commencement and throughout its progress. In an address I gave in 1891, "on some points of recent treatment in the deformities of children," I concluded by emphasising the fact, and I cannot do better than repeat it here—that the more attention we pay to these little patients with deformities such as I have mentioned, the earlier they are treated, and the more systematic the examination, the greater will be our success in alleviating and perhaps removing conditions which would prevent such patients leading an active and useful life.

